# Patient-Reported Burden and Clinical Spectrum of Rheumatic Immune-Related Adverse Events After Immune Checkpoint Inhibitor Therapy in Lung Cancer: A Real-World Cohort Study

**DOI:** 10.3390/curroncol33100603

**Published:** 2026-10-07

**Authors:** Zhenhong Ren, Jianchao Lu

**Affiliations:** 1 Department of Pharmacy, Sichuan Clinical Research Center for Cancer, Sichuan Cancer Hospital & Institute, Sichuan Cancer Center, University of Electronic Science and Technology of China, No. 55, Section 4, South Renmin Road, Chengdu 610041, China; renzhenhong@scszlyy.org.cn; 2 Department of Radiation Oncology, Precision Radiation in Oncology Key Laboratory of Sichuan Province, Sichuan Clinical Research Center for Cancer, Sichuan Cancer Hospital & Institute, Sichuan Cancer Center, University of Electronic Science and Technology of China, No. 55, Section 4, South Renmin Road, Chengdu 610041, China

**Keywords:** immune checkpoint inhibitor, rheumatic immune-related adverse event, lung cancer, inflammatory arthritis, patient-reported outcome, C-reactive protein

## Abstract

Background: Rheumatic immune-related adverse events (R-irAEs) after immune checkpoint inhibitor (ICI) therapy in lung cancer can be difficult to distinguish from cancer-related or degenerative musculoskeletal symptoms. Methods: In this single-center retrospective cohort, we analyzed 768 ICI-treated patients and a nested questionnaire cohort to characterize rheumatologist-confirmed R-irAEs, baseline associated factors, and patient-reported outcomes. Missing baseline C-reactive protein (CRP) was handled using multiple imputation. Results: New musculoskeletal symptoms occurred in 177 patients (23.0%), and 66 patients (8.6%) had confirmed R-irAEs. The leading phenotypes were inflammatory arthritis (*n* = 22), inflammatory arthralgia (*n* = 15), and polymyalgia rheumatica-like syndrome (*n* = 13). In multivariable analysis, female sex (adjusted odds ratio [OR] 2.21, 95% confidence interval [CI] 1.31–3.74), baseline CRP > 10 mg/L (OR 2.08, 95% CI 1.14–3.81), and pre-existing autoimmune disease (OR 5.24, 95% CI 2.37–11.57) were associated with confirmed R-irAEs. Among 437 questionnaire responders, confirmed R-irAEs were associated with worse pain, morning stiffness, disability, fatigue, and health status than non-confirmed musculoskeletal symptoms (all Holm-adjusted *p* < 0.001). Conclusions: Confirmed R-irAEs affected a measurable minority of ICI-treated patients with lung cancer and were associated with worse post-treatment patient-reported outcomes. Because baseline patient-reported outcomes were unavailable, these findings should not be interpreted as R-irAE-attributable change.

## Simple Summary

Some patients with lung cancer develop new joint, muscle, or dryness symptoms after immune checkpoint inhibitor therapy, but these symptoms are not always caused by immune-mediated rheumatic disease. In this real-world cohort, 8.6% of patients had rheumatologist-confirmed rheumatic immune-related adverse events, most often inflammatory arthritis, inflammatory arthralgia, or polymyalgia rheumatica-like syndrome. Among questionnaire responders, patients with confirmed events reported greater pain, longer morning stiffness, greater functional disability, more fatigue, and lower health status than patients with non-confirmed musculoskeletal symptoms. Female sex, elevated baseline C-reactive protein, and pre-existing autoimmune disease were associated with confirmed events, although these findings require external validation. Because baseline patient-reported outcomes were unavailable, these comparisons describe post-treatment symptom differences rather than change attributable to R-irAEs. These results support risk-aware symptom surveillance and early rheumatology–oncology collaboration when inflammatory features are present.

## 1. Introduction

Immune checkpoint inhibitors have become a central component of lung cancer treatment across metastatic, locally advanced, and perioperative settings. In non-small-cell lung cancer, PD-1/PD-L1-directed therapy has improved outcomes as monotherapy in selected PD-L1-positive disease, in combination with chemotherapy, and after definitive chemoradiotherapy for unresectable stage III disease [1,2,3,4,5]. Wider ICI use has also expanded the population at risk for immune-mediated toxicity, including patients with multiple comorbidities and patients treated outside the selection boundaries of pivotal trials [6,7].

Immune-related adverse events (irAEs) reflect immune activation against non-tumor tissues and may involve almost any organ system [6,8]. Oncology guidelines now provide practical severity-based approaches to common irAEs, but musculoskeletal and rheumatic presentations remain less standardized than endocrine, dermatologic, gastrointestinal, or pulmonary toxicities [9,10]. The EULAR points to consider emphasize early recognition, exclusion of mimicking conditions, and multidisciplinary decision-making because rheumatic symptoms may be treatable without automatically stopping effective cancer therapy [11].

Rheumatic irAEs occupy a clinically awkward space. They include inflammatory arthritis, inflammatory arthralgia, PMR-like syndrome, sicca manifestations, myositis, vasculitis, and spondyloarthritis-like disease, but the boundary between immune-mediated disease and non-inflammatory pain is often blurred [12,13,14]. In a single-center prospective cohort, rheumatic disorders associated with ICIs were heterogeneous and appeared to require rheumatology input for phenotype-level diagnosis and management [15]. Subsequent series have shown that ICI-induced inflammatory arthritis may differ by immunotherapy regimen and can persist beyond ICI cessation in some patients [16,17].

For patients with lung cancer, musculoskeletal symptoms are especially challenging to interpret. Bone metastases, degenerative disease, paraneoplastic symptoms, treatment-related fatigue, and frailty may all coexist with ICI exposure. Large cohorts suggest that musculoskeletal irAEs are frequent but often mild, whereas true inflammatory rheumatic phenotypes are less common and require more careful attribution [18,19]. Sicca syndrome and PMR-like presentations have been described as recognizable ICI-associated syndromes, but they do not always map neatly onto classical Sjögren disease or primary PMR [20,21,22,23].

Risk stratification also remains unsettled. Pre-existing autoimmune disease increases concern for flare or de novo irAEs, yet observational studies indicate that ICIs can often still be used when cancer benefit is compelling and monitoring is appropriate [24,25,26]. Baseline systemic inflammation, including C-reactive protein (CRP), has been evaluated as a possible marker of irAE risk in non-small-cell lung cancer, although its biological interpretation is complicated by cancer burden, infection, and comorbidity [27]. Sex-related differences in ICI toxicity are biologically plausible but inconsistent across large datasets and meta-analyses [28,29].

Most existing reports focus on clinician-coded toxicity, phenotype description, or treatment. Fewer studies quantify patient-reported pain, stiffness, disability, and health status while also separating rheumatologist-confirmed R-irAEs from non-confirmed post-ICI musculoskeletal symptoms. This distinction is important because a comparator group consisting only of patients without R-irAEs can hide the clinical problem faced in practice: deciding which symptomatic patients have immune-mediated rheumatic disease and how much burden confirmed disease adds. We therefore studied an institutional lung cancer ICI cohort with rheumatologist-confirmed R-irAE adjudication, baseline risk-factor analysis, and a nested questionnaire assessment of symptom burden.

## 2. Materials and Methods

### 2.1. Study Design and Population

This single-center retrospective observational cohort study included patients with lung cancer who received immune checkpoint inhibitor (ICI)-based treatment at Sichuan Cancer Hospital and Institute between September 2023 and December 2025. The source population comprised 768 patients in the institutional lung cancer ICI analysis dataset. Patients were classified according to post-ICI musculoskeletal status into three mutually exclusive groups: no new musculoskeletal (MSK) symptom after ICI initiation, non-confirmed MSK symptom, and rheumatologist-confirmed rheumatic immune-related adverse event (R-irAE). A nested questionnaire analysis was further conducted among patients who completed the patient-reported outcome survey, including respondents from all three post-ICI musculoskeletal-status groups.

This manuscript was prepared in accordance with the Strengthening the Reporting of Observational Studies in Epidemiology (STROBE) reporting guideline; the completed checklist is provided as a non-published reporting checklist.

### 2.2. Definition and Clinical Classification of R-irAEs

The primary clinical outcome was rheumatologist-confirmed R-irAE after ICI initiation. New post-ICI musculoskeletal symptoms were identified from the institutional dataset and all symptomatic cases underwent rheumatologist adjudication using the available clinical record and clinical attribution. Patients were classified as having non-confirmed MSK symptoms or confirmed R-irAEs; patients without a new post-ICI MSK symptom comprised the third group. Formal rheumatology referral during routine care was recorded separately from adjudication. Patients who were formally referred underwent clinical rheumatology assessment, whereas cases without a formal referral could be adjudicated retrospectively by rheumatologist review of the medical record. R-irAE phenotype was assigned as inflammatory arthritis, inflammatory arthralgia, PMR-like syndrome, sicca syndrome, myositis, spondyloarthritis-like disease, or vasculitis/overlap.

R-irAE onset was defined as the number of days from ICI initiation to documented onset of the confirmed rheumatic event. Severity was graded using the Common Terminology Criteria for Adverse Events, version 5.0 [30]. Management variables included formal rheumatology referral, systemic glucocorticoid use, conventional synthetic disease-modifying antirheumatic drug (csDMARD) use, biologic DMARD use, temporary ICI hold, permanent ICI discontinuation, and documented improvement or resolution. For reporting, temporary ICI hold and permanent ICI discontinuation were treated as mutually exclusive categories; patients who initially had ICI treatment held and subsequently permanently discontinued ICI because of R-irAE were classified in the permanent-discontinuation category only. For sicca syndrome, improvement was defined as documented symptomatic improvement based on patient report and/or treating clinician assessment and did not require complete resolution or objective recovery of salivary or lacrimal gland function.

### 2.3. Baseline Variables and Laboratory Workup

Baseline variables included age, sex, body mass index, smoking status, ECOG PS, histology, stage group, metastatic sites, PD-L1 tumor proportion score group, actionable driver status, treatment setting/line, ICI regimen and class, pre-existing autoimmune history, baseline CRP, and baseline eosinophil count. Pre-existing autoimmune disease was coded as any baseline autoimmune history other than “no autoimmune disease.” Baseline CRP was categorized as >10 mg/L versus ≤10 mg/L; missing baseline CRP was handled as described in the statistical analysis. ICI plus chemotherapy was defined from the regimen variable.

Among confirmed R-irAE cases, workup availability was summarized for clinically relevant denominators. CRP and erythrocyte sedimentation rate (ESR) peaks, creatine kinase testing, troponin testing for myositis, and autoantibody testing were recorded when available. Autoantibodies included antinuclear antibody, rheumatoid factor, anti-cyclic citrullinated peptide antibody, and SSA/SSB antibodies. Autoantibody testing was also summarized among patients with non-confirmed MSK symptoms when clinically obtained. Because testing was clinically driven, denominators reflect test availability and positivity was not interpreted as a prevalence estimate.

### 2.4. Nested Questionnaire Cohort and Patient-Reported Outcomes

The nested questionnaire cohort included patients who completed the questionnaire from the three musculoskeletal symptom groups. Patient-reported outcomes included pain visual analog scale (VAS, 0–10), morning stiffness duration, the Health Assessment Questionnaire Disability Index (HAQ-DI, 0–3), EQ-5D-5L index, and Functional Assessment of Chronic Illness Therapy-Fatigue (FACIT-F) score. HAQ-DI was used as a functional-disability measure grounded in the Health Assessment Questionnaire framework [31,32]. EQ-5D-5L was used to summarize health status [33]. FACIT-F was used to capture fatigue and its impact on daily activity and function [34].

The individual patient-reported measures were treated as the primary patient-reported outcomes. As a secondary exploratory summary, an analysis-defined rheumatic symptom-burden score was constructed on a 0–100 scale, with higher values indicating greater burden. The score was calculated as 100 × [0.35 × pain VAS/10 + 0.25 × HAQ-DI/3 + 0.20 × (52 − FACIT-F)/52 + 0.20 × min(morning stiffness in minutes, 120)/120]. Only respondents with complete data for all four components contributed to the composite analysis. The score was not considered a separately validated instrument. Sensitivity analyses evaluated equal weighting and alternative one-domain-emphasis weighting schemes to assess dependence on the original weights.

### 2.5. Exploratory Tumor Outcomes

Objective response, disease control, progression-free survival (PFS), and overall survival (OS) were summarized descriptively by confirmed R-irAE status. Because R-irAE is a post-baseline event, tumor-outcome analyses were considered exploratory and were not used to infer that R-irAE independently predicts treatment benefit. To reduce immortal-time bias in exploratory PFS analyses, a 3-month landmark analysis and a time-dependent Cox model were performed.

### 2.6. Statistical Analysis

Continuous variables were summarized as medians and interquartile ranges (IQRs), and categorical variables as counts and percentages. Baseline characteristics were compared between patients with and without confirmed R-irAEs using Wilcoxon rank-sum tests, Pearson chi-square tests, or Fisher exact tests, as appropriate. Patient-reported measures across the three musculoskeletal symptom groups were compared using Kruskal–Wallis tests. Adjusted comparisons for each individual patient-reported outcome were estimated using multivariable linear regression with HC3 robust standard errors; Holm adjustment was applied across the 10 group contrasts.

A core multivariable logistic regression model was used to evaluate baseline factors associated with confirmed R-irAE. The model included female sex, age, ECOG PS ≥ 2, ICI plus chemotherapy, baseline CRP > 10 mg/L, and pre-existing autoimmune disease. The reduced covariate set was used to limit overfitting given 66 confirmed events. Missing baseline CRP (103/768, 13.4%) was handled using multiple imputation by chained equations with 50 imputations; continuous CRP was imputed using predictive mean matching and the >10 mg/L indicator was then derived within each imputed dataset. Estimates were pooled using Rubin’s rules. Complete-case logistic regression, complete-case Firth logistic regression, and a definite-only R-irAE Firth model were used as sensitivity analyses. The results are reported as adjusted odds ratios (ORs) and 95% confidence intervals (CIs). The CRP imputation model included the R-irAE outcome, all non-CRP core-model covariates, and auxiliary baseline variables (body mass index, stage IV/extensive disease, bone and brain metastases, albumin, hemoglobin, neutrophil, lymphocyte, and platelet counts).

For each individual patient-reported outcome, multivariable linear regression was adjusted for age, sex, ECOG PS ≥ 2, stage IV/extensive disease, bone metastasis, and pre-existing autoimmune disease. A secondary sensitivity analysis used inverse probability weighting (IPW) to account for questionnaire response among questionnaire-eligible patients. The response model included age, sex, ECOG PS ≥ 2, stage IV/extensive disease, bone metastasis, pre-existing autoimmune disease, confirmed R-irAE, and new musculoskeletal symptoms after ICI; stabilized weights were truncated at the 1st and 99th percentiles. The secondary composite score was evaluated under the original weighting scheme and five alternative weighting schemes. Analyses were conducted in R using tidyverse, gtsummary, flextable, officer, survival, survminer, broom, gridExtra, mice, logistf, sandwich, lmtest, and survey packages. Two-sided *p* values < 0.05 were considered statistically significant. For response-bias analyses, questionnaire eligibility required being alive at the questionnaire assessment window; 762 of 768 patients met this criterion.

## 3. Results

### 3.1. Cohort Assembly

Among 768 patients with lung cancer treated with ICIs, 177 (23.0%) developed new musculoskeletal symptoms after ICI initiation. Rheumatologist-confirmed R-irAEs were identified in 66 patients (8.6% of the parent cohort), and 111 patients (14.5%) had new musculoskeletal symptoms that were not confirmed as R-irAEs. The nested questionnaire cohort included 437 responders: 318 without new musculoskeletal symptoms, 68 with non-confirmed musculoskeletal symptoms, and 51 with confirmed R-irAEs (Figure 1).

### 3.2. Baseline Characteristics

The median age of the cohort was 63.0 years (IQR 57.0–69.0), and 299 patients (38.9%) were female. Adenocarcinoma was the most frequent histology (515/768, 67.1%), and 558 patients (72.7%) had stage IV/extensive disease. ICI plus chemotherapy was the most common regimen (418/768, 54.4%). PD-1-based therapy accounted for most treatment exposure.

Compared with patients without confirmed R-irAEs, those with confirmed R-irAEs were more often female (57.6% vs. 37.2%, *p* = 0.001), more frequently had a baseline autoimmune history (16.7% vs. 3.6%, *p* < 0.001), and had a higher baseline CRP (12.3 mg/L [IQR 7.5–17.9] vs. 9.1 mg/L [IQR 5.8–14.7], *p* = 0.003). Age, body mass index, smoking status, ECOG PS, histology, stage group, bone or brain metastasis, PD-L1 tumor proportion score, driver status, treatment setting/line, ICI regimen, ICI class, and baseline eosinophil count did not differ materially between groups in unadjusted comparisons (Table 1).

### 3.3. Clinical Spectrum, Onset, and Management of Confirmed R-irAEs

Ten confirmed cases (15.2%) were grade ≥ 3. Grade ≥ 3 events were observed in inflammatory arthritis (5/22), PMR-like syndrome (3/13), inflammatory arthralgia (1/15), and myositis (1/4). Overall, 51 patients (77.3%) had a formal rheumatology referral, 36 (54.5%) received systemic glucocorticoids, and 14 (21.2%) received a csDMARD. Regarding ICI management, 21 patients (31.8%) had treatment temporarily held without subsequent permanent discontinuation, whereas five patients (7.6%) permanently discontinued ICI because of R-irAE; four of the latter had initially had treatment held. Improvement or resolution was documented in 62 patients (93.9%) (Table 2; Figure 2).

Ten confirmed cases (15.2%) were grade ≥ 3. Grade ≥ 3 events were observed in inflammatory arthritis (5/22), PMR-like syndrome (3/13), inflammatory arthralgia (1/15), and myositis (1/4). Overall, 51 patients (77.3%) had a formal rheumatology referral, 36 (54.5%) received systemic glucocorticoids, 14 (21.2%) received a csDMARD, and none received a biologic DMARD. ICI was temporarily held in 25 patients (37.9%) and permanently discontinued in five (7.6%) because of R-irAE. Improvement or resolution was documented in 62 patients (93.9%). Among the eight sicca cases, all had documented symptomatic improvement during follow-up, but none had documented complete resolution; hydroxychloroquine was used in two patients and methotrexate in one for concomitant inflammatory arthralgia/arthritis rather than for sicca itself. Among the four myositis cases, no myocarditis or myasthenic syndrome was documented.

Workup data were complete for R-irAE onset date, CTCAE grade, rheumatology assessment delay, peak CRP, and peak ESR among confirmed cases. Autoantibody testing among confirmed cases was performed in 58 patients for ANA, 56 for rheumatoid factor, 53 for anti-CCP, and 21 for SSA/SSB; positivity among tested patients was 36.2%, 12.5%, 3.8%, and 9.5%, respectively. Among the 111 patients with non-confirmed MSK symptoms, ANA, rheumatoid factor, and anti-CCP were clinically obtained in 24, 14, and 14 patients, respectively, and SSA/SSB testing was not recorded. Because testing was clinically driven and denominators differed substantially, these positivity proportions were not interpreted as prevalence estimates (Supplementary Table S8).

### 3.4. Baseline Factors Associated with Confirmed R-irAEs

In the multiple-imputation core multivariable logistic model, female sex was associated with confirmed R-irAE (adjusted OR 2.21, 95% CI 1.31–3.74; *p* = 0.003). Baseline CRP > 10 mg/L was also associated with confirmed R-irAE (adjusted OR 2.08, 95% CI 1.14–3.81; *p* = 0.017), as was pre-existing autoimmune disease (adjusted OR 5.24, 95% CI 2.37–11.57; *p* < 0.001). Age, ECOG PS ≥ 2, and ICI plus chemotherapy were not statistically significant in this model (Supplementary Table S4; Supplementary Figure S1).

Complete-case logistic and Firth logistic analyses were directionally consistent with the primary multiple-imputation model. In the definite-only R-irAE Firth analysis, baseline CRP >10 mg/L (OR 2.39, 95% CI 1.23–4.85; *p* = 0.010) and pre-existing autoimmune disease (OR 4.87, 95% CI 1.85–11.67; *p* = 0.002) remained associated with the outcome, whereas the female-sex association was attenuated (OR 1.67, 95% CI 0.87–3.21; *p* = 0.121) (Supplementary Table S5).

### 3.5. Patient-Reported Symptom Burden

Among 437 questionnaire responders, patients with confirmed R-irAEs had worse patient-reported outcomes across all five individual domains. Median pain VAS was 5.1 (IQR 4.0–6.3) in the confirmed R-irAE group, compared with 3.5 (IQR 2.3–4.5) in the non-confirmed MSK symptom group and 1.9 (IQR 0.8–3.1) in those without new MSK symptoms (*p* < 0.001). Median morning stiffness was 37.5 min (IQR 25.0–64.0), 8.0 min (IQR 5.0–14.0), and 3.0 min (IQR 1.0–6.0), respectively (*p* < 0.001). HAQ-DI was higher, FACIT-F was lower, and EQ-5D-5L index was lower in the confirmed R-irAE group than in the other groups (Table 3; Figure 3).

Nested questionnaire cohort only. Values in Panel A are median (IQR), with the valid *n* for each measure shown within cells because of item-level missingness. Higher pain VAS, morning stiffness, and HAQ-DI indicate worse status; higher FACIT-F and EQ-5D-5L indicate better status. Descriptive *p* values compare the three symptom groups using Kruskal–Wallis tests. Adjusted mean differences were estimated using multivariable linear regression with HC3 robust standard errors and adjustment for age, sex, ECOG PS ≥ 2, stage IV/extensive disease, bone metastasis, and pre-existing autoimmune disease. Holm-adjusted *p* values account for the 10 reported group contrasts. MSK, musculoskeletal; VAS, visual analog scale; HAQ-DI, Health Assessment Questionnaire Disability Index; FACIT-F, Functional Assessment of Chronic Illness Therapy-Fatigue.

After adjustment for age, sex, ECOG PS ≥ 2, stage IV/extensive disease, bone metastasis, and pre-existing autoimmune disease, confirmed R-irAE was associated with worse outcomes than non-confirmed MSK symptoms: pain VAS was 1.8 points higher (95% CI 1.2–2.4), morning stiffness was 40.5 min longer (95% CI 29.1–51.9), HAQ-DI was 0.30 higher (95% CI 0.20–0.41), FACIT-F was 7.8 points lower (95% CI −10.2 to −5.4), and EQ-5D-5L index was 0.117 lower (95% CI −0.155 to −0.080); all Holm-adjusted *p* < 0.001. Contrasts with the no new MSK symptom group were also statistically significant (Table 3). As a secondary exploratory analysis among 396 respondents with complete data for all four composite components, the original-weight composite was 18.4 points higher in confirmed R-irAEs than in non-confirmed MSK symptoms (95% CI 14.5–22.4) and 26.9 points higher than in those without new MSK symptoms (95% CI 23.4–30.3). The direction and statistical significance were unchanged across five alternative weighting schemes (Supplementary Table S7).

Among 762 questionnaire-eligible patients, responders were more likely than eligible nonresponders to have confirmed R-irAEs (11.7% vs. 4.6%, *p* < 0.001) and new post-ICI MSK symptoms (27.2% vs. 17.5%, *p* = 0.002). Stabilized and truncated IPW estimates for the five individual patient-reported outcomes were very similar to the primary adjusted estimates (Supplementary Tables S2 and S9). Because response was more frequent among patients with confirmed R-irAEs, the absolute symptom burden observed in the responder cohort may be upwardly biased despite IPW adjustment.

### 3.6. Exploratory Tumor Outcomes

Unadjusted objective response and disease control were 53.0% and 89.4% among patients with confirmed R-irAEs, compared with 44.7% and 83.9% among those without confirmed R-irAEs. Median PFS was 12.2 months (IQR 8.1–19.1) in the confirmed R-irAE group and 8.4 months (IQR 4.1–15.3) in the no confirmed R-irAE group; median OS was 19.9 months (IQR 13.3–26.3) and 20.9 months (IQR 14.4–28.5), respectively. Because R-irAE occurred after treatment initiation, these unadjusted differences were treated as descriptive only. The 3-month landmark PFS model did not show a statistically significant association for early R-irAE (adjusted HR 0.79, 95% CI 0.51–1.22; *p* = 0.283). The time-dependent Cox model yielded an adjusted HR of 0.75 (95% CI 0.56–1.00; *p* = 0.049), but this analysis was exploratory and was not interpreted as evidence that R-irAE independently predicts tumor benefit.

## 4. Discussion

This study adds a patient-centered layer to the clinical description of rheumatic toxicity after ICI therapy in lung cancer. In 768 ICI-treated patients, nearly one quarter developed new musculoskeletal symptoms, but fewer than half of these symptomatic patients had rheumatologist-confirmed R-irAEs. This separation matters: in practice, a new pain syndrome in a patient with lung cancer may reflect immune-mediated disease, cancer-related symptoms, degenerative disease, treatment fatigue, or several of these at once. Reports based only on adverse-event coding can miss that ambiguity [12,18]. Recent prospective whole-body MRI data have also shown inflammatory lesions in ICI-exposed patients presenting with arthralgia as well as inflammatory arthritis, reinforcing the difficulty of phenotype attribution from symptoms alone [35].

The confirmed R-irAE rate of 8.6% sits within the broad range expected for clinically meaningful rheumatic toxicity, while the 23.0% rate of new musculoskeletal symptoms illustrates why careful attribution is needed. Trial-level meta-analyses have consistently shown that arthralgia and myalgia are among the more frequent musculoskeletal complaints during ICI therapy, but true inflammatory rheumatic disease is less consistently captured because trial reporting often emphasizes higher-grade events [12,19]. In the 927-patient solid-tumor cohort reported by Melia and colleagues, musculoskeletal irAEs were frequent and mostly mild, a pattern that is compatible with our finding that many post-ICI musculoskeletal symptoms were not ultimately confirmed as R-irAEs [18].

The phenotype distribution in our cohort is clinically recognizable: inflammatory arthritis, inflammatory arthralgia, and PMR-like syndrome accounted for most confirmed cases. This pattern agrees with early rheumatology case series and prospective cohorts, which identified inflammatory arthritis and PMR-like syndromes as prominent ICI-associated presentations [13,15]. The relatively small number of myositis cases is also consistent with its lower frequency but higher clinical stakes, particularly because ICI-associated myositis can overlap with myocarditis or myasthenia-like syndromes and often warrants urgent evaluation rather than routine outpatient triage [10,36].

Onset timing differed by phenotype. Myositis appeared early, with a median onset of less than one month, while sicca manifestations tended to appear later. The early onset of ICI myositis is clinically important because delayed recognition can be dangerous when cardiac or neuromuscular overlap is present [10,36]. Sicca syndrome has been described as an ICI-associated condition distinct from classic Sjögren disease in many patients, with salivary gland injury and variable recovery [20]. Our later median onset for sicca should be read cautiously because only eight cases were included, but it supports continued symptom surveillance beyond the first few cycles of ICI therapy. In our four myositis cases, no myocarditis or myasthenic syndrome was documented. Among the eight sicca cases, all had documented symptomatic improvement during follow-up, but none had documented complete resolution or objective recovery of salivary or lacrimal gland function.

The PMR-like cases also deserve comment. ICI-associated PMR may resemble primary PMR but can differ in inflammatory intensity, imaging findings, and treatment requirements [21,22]. A recent comparison of ICI-mediated PMR with primary PMR suggested that ICI-PMR may have a milder course and less intense inflammation in some patients [23]. Our PMR-like group had a median onset near 3.5 months, substantial glucocorticoid use, and a grade ≥ 3 proportion similar to inflammatory arthritis, emphasizing that “PMR-like” should not be treated as a minor label when symptoms limit function.

The risk-factor findings are plausible, but they should not be oversold. Pre-existing autoimmune disease showed the strongest association with confirmed R-irAE. That observation fits the broader literature: patients with autoimmune disease were often excluded from ICI trials, yet observational data show higher rates of autoimmune flare or irAEs while also suggesting that ICIs can remain feasible with careful selection and monitoring [24,25,26]. Our result supports heightened vigilance rather than automatic exclusion from ICI treatment. The autoimmune disease estimate remained imprecise (95% CI 2.37–11.57) and, given only 66 confirmed events, should be viewed as hypothesis-generating rather than as a definitive risk estimate.

Female sex was associated with confirmed R-irAE in this cohort. The finding is biologically credible given the female predominance of many systemic autoimmune diseases, but ICI toxicity studies have not reached a uniform conclusion on sex effects. Jing and colleagues reported minimal sex-associated differences in irAE risk across large datasets [28]. A more recent meta-analysis of clinical trials also found that sex-related toxicity patterns are complex and not easily reduced to a single global risk estimate [29]. For that reason, we frame the female-sex association as a cohort finding that should guide awareness and future validation, not as a definitive screening rule. The association with female sex was attenuated in the definite-only sensitivity analysis (OR 1.67, 95% CI 0.87–3.21), which further supports cautious interpretation.

Baseline CRP > 10 mg/L was also associated with confirmed R-irAE. This is one of the more interesting findings because CRP is both clinically available and biologically nonspecific. In non-small-cell lung cancer, Onodera and colleagues evaluated pretreatment CRP using a 10 mg/L cutoff and reported an association with irAE incidence [27]. CRP may reflect a baseline inflammatory state that facilitates immune toxicity, but in lung cancer it may also reflect tumor burden, infection, chronic lung disease, or tissue injury. The primary analysis used multiple imputation for the 13.4% of patients with missing baseline CRP, and complete-case and Firth analyses were directionally consistent. Nevertheless, nonrandom CRP measurement and residual confounding remain possible, so the CRP association should be considered exploratory rather than a screening marker.

Autoantibody results in our cohort were not strongly suggestive of classical seropositive rheumatic disease. Anti-CCP positivity was rare, rheumatoid factor positivity occurred in a minority of tested patients, and SSA/SSB positivity was uncommon among those tested. This pattern is consistent with the literature showing that ICI-induced inflammatory arthritis is often seronegative and may differ immunologically from classical rheumatoid arthritis [16,37]. At the same time, pre-ICI rheumatoid factor has been associated with higher odds of rheumatic irAEs in selected tested populations [38]. The practical message is not to screen every patient indiscriminately; rather, autoantibodies should be interpreted in clinical context when symptoms or autoimmune history make testing relevant [11]. In the present cohort, testing was clinically driven and occurred much more frequently among confirmed R-irAE cases than among patients with non-confirmed MSK symptoms; accordingly, positivity proportions were summarized among tested patients and were not treated as prevalence estimates.

The main contribution of this study is the nested patient-reported analysis. Confirmed R-irAE patients reported higher pain, longer morning stiffness, greater HAQ-DI disability, more fatigue as reflected by lower FACIT-F scores, and lower EQ-5D-5L health status. Importantly, the comparison was not limited to asymptomatic patients: confirmed R-irAEs also had worse adjusted outcomes than non-confirmed post-ICI MSK symptoms across all five individual domains. This design addresses the clinically relevant distinction between nonspecific post-treatment symptoms and rheumatologist-confirmed inflammatory toxicity.

The individual patient-reported differences were similar after IPW adjustment for questionnaire response. The secondary analysis-defined composite also showed the same direction of association across six weighting schemes, indicating that the composite result was not dependent on a single weighting recipe. However, IPW cannot eliminate bias from unmeasured determinants of response, and the higher questionnaire response among patients with confirmed R-irAEs means that absolute symptom burden may be overestimated. In addition, because patient-reported outcomes were not collected before ICI initiation, the post-treatment differences cannot be separated completely from pre-existing symptoms or interpreted as within-patient change caused by R-irAEs.

Management patterns were broadly consistent with guideline principles. More than half of confirmed cases received systemic glucocorticoids, about one fifth received a csDMARD, no patient received a biologic DMARD, and temporary ICI hold was more common than permanent discontinuation. EULAR and ASCO guidance both support severity-adapted management, multidisciplinary discussion, and attempts to control rheumatic toxicity without unnecessarily compromising cancer therapy [9,10,11]. The high rate of documented improvement or resolution in our cohort is reassuring, but it should not be interpreted as evidence that these events are trivial. For sicca specifically, improvement referred to symptomatic improvement rather than complete or objectively documented gland recovery. Recent observational data also show that the course and resolution of ICI-induced inflammatory arthritis vary during ongoing ICI treatment and after ICI discontinuation [39].

The exploratory oncology outcomes should be interpreted with restraint. Patients with confirmed R-irAEs had higher unadjusted response and PFS descriptively, and the time-dependent model suggested a lower PFS hazard, whereas the 3-month landmark model did not reach statistical significance. Many studies have linked irAEs with better ICI outcomes, including NSCLC cohorts [40,41]. However, such associations are vulnerable to immortal-time bias, treatment-duration bias, surveillance intensity, and confounding by patients living long enough to develop toxicity. Our analysis was not designed to establish an antitumor benefit of R-irAE, and the manuscript deliberately avoids that claim.

This study has several strengths. It focuses on lung cancer, uses rheumatologist-confirmed R-irAE rather than adverse-event codes alone, separates confirmed R-irAEs from non-confirmed post-ICI musculoskeletal symptoms, and includes a nested questionnaire assessment that captures the patient’s pain, stiffness, function, fatigue, and health status. The use of adjusted and sensitivity analyses makes the central burden finding less dependent on any single modeling choice.

The limitations are equally important. The study was retrospective, observational, and single-center, so external validation is needed. Because the cohort was drawn from a single Chinese cancer center, with a relatively high never-smoker fraction and a low proportion of actionable-driver-positive disease, generalizability to populations with different demographic, smoking, molecular, or treatment profiles may be limited. Formal rheumatology referral and the timing and intensity of clinical assessment were not protocolized, although R-irAE status was adjudicated by rheumatologist review. Baseline CRP was missing in 13.4% of patients; multiple imputation reduced reliance on complete cases but cannot exclude bias if missingness was not adequately captured by the imputation model. Autoantibody testing was clinically driven rather than protocolized and therefore cannot provide prevalence estimates. Rare phenotypes such as myositis, spondyloarthritis-like disease, and vasculitis/overlap had small numbers, so their onset and management estimates are descriptive. Patient-reported outcomes were collected after ICI initiation without baseline measurements, limiting separation of R-irAE-associated burden from pre-existing symptoms. The composite symptom-burden score was analysis-defined, was used only as a secondary exploratory measure, and is not a validated standalone instrument. Finally, questionnaire response was more frequent among patients with confirmed R-irAEs; IPW produced similar estimates but cannot fully eliminate response bias, and absolute burden in responders may therefore be upwardly biased.

## 5. Conclusions

In summary, rheumatologist-confirmed R-irAEs occurred in a measurable minority of ICI-treated patients with lung cancer and were associated with worse post-treatment patient-reported outcomes than non-confirmed MSK symptoms. These observational findings support risk-aware symptom surveillance, early rheumatology involvement when inflammatory features are present, and prospective integration of baseline and longitudinal patient-reported outcomes into ICI toxicity monitoring.

## Figures and Tables

**Figure 1:**
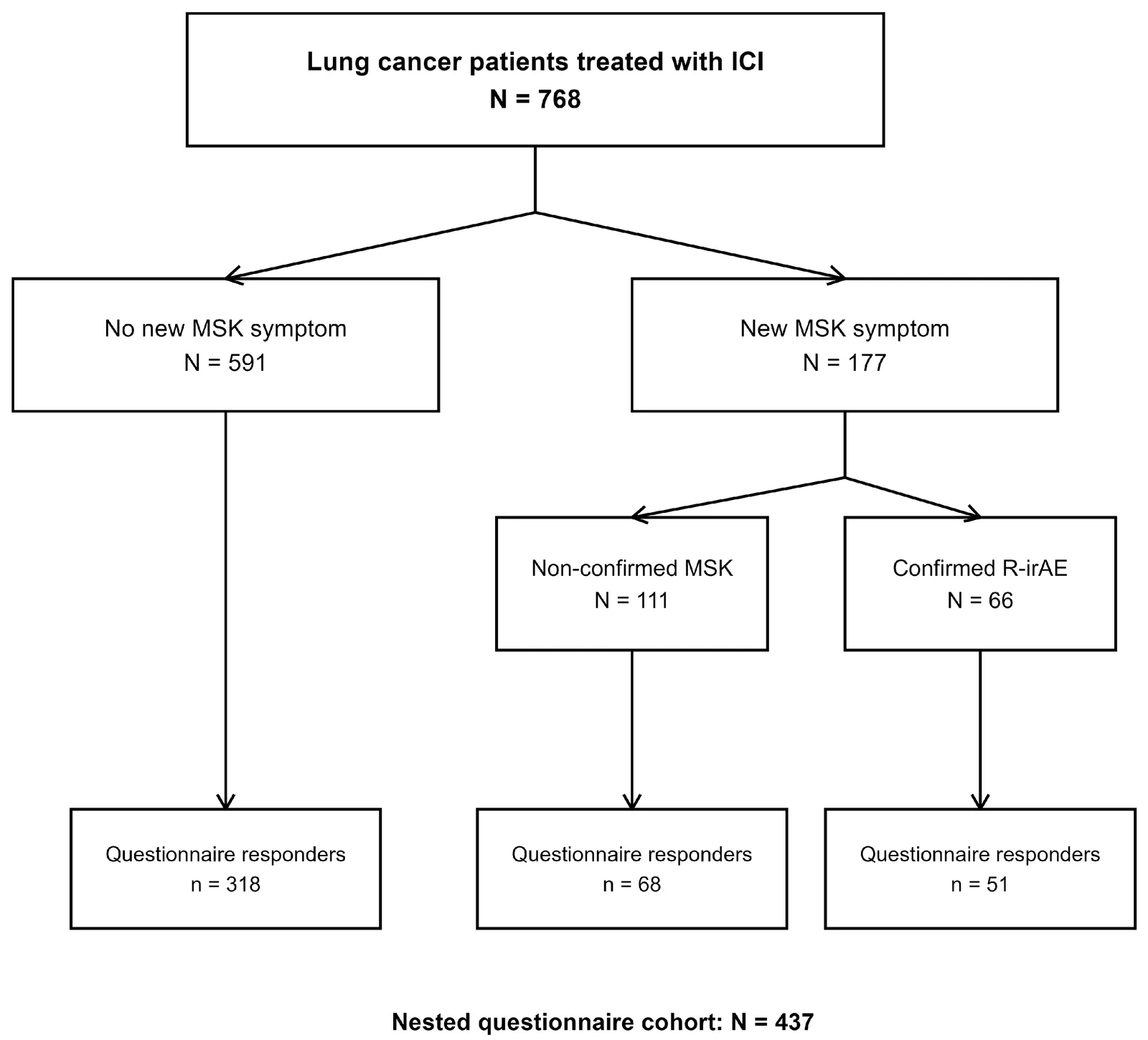
Study cohort and nested questionnaire flow. The flow diagram shows the assembly of the parent lung cancer immune checkpoint inhibitor (ICI) cohort and the nested questionnaire cohort. Among 768 lung cancer patients treated with ICIs, 177 developed new musculoskeletal (MSK) symptoms after ICI initiation and were classified as non-confirmed MSK symptoms or rheumatologist-confirmed rheumatic immune-related adverse events (R-irAEs). Questionnaire responders are shown separately within each of the three post-ICI musculoskeletal-status groups. ICI, immune checkpoint inhibitor; MSK, musculoskeletal; R-irAE, rheumatic immune-related adverse event.

**Figure 2:**
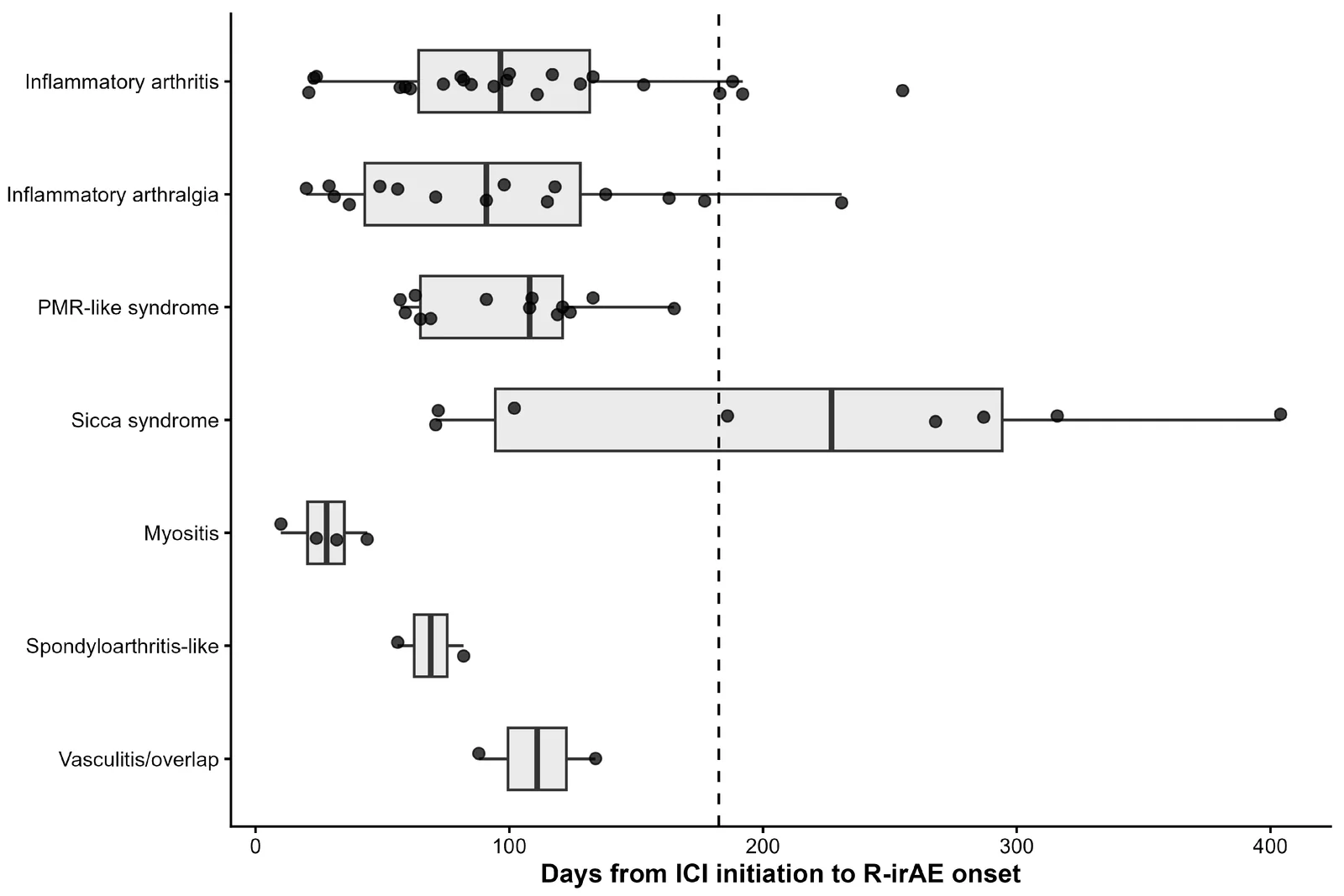
Time to onset of confirmed rheumatic irAEs by clinical phenotype. The plot shows the time from ICI initiation to onset of rheumatologist-confirmed R-irAEs according to clinical phenotype. Each point represents one confirmed R-irAE case, and box plots summarize the distribution within each phenotype. The dashed vertical line indicates 6 months after ICI initiation. Myositis tended to occur earlier after ICI exposure, whereas sicca manifestations generally appeared later. ICI, immune checkpoint inhibitor; R-irAE, rheumatic immune-related adverse event; PMR, polymyalgia rheumatica.

**Figure 3:**
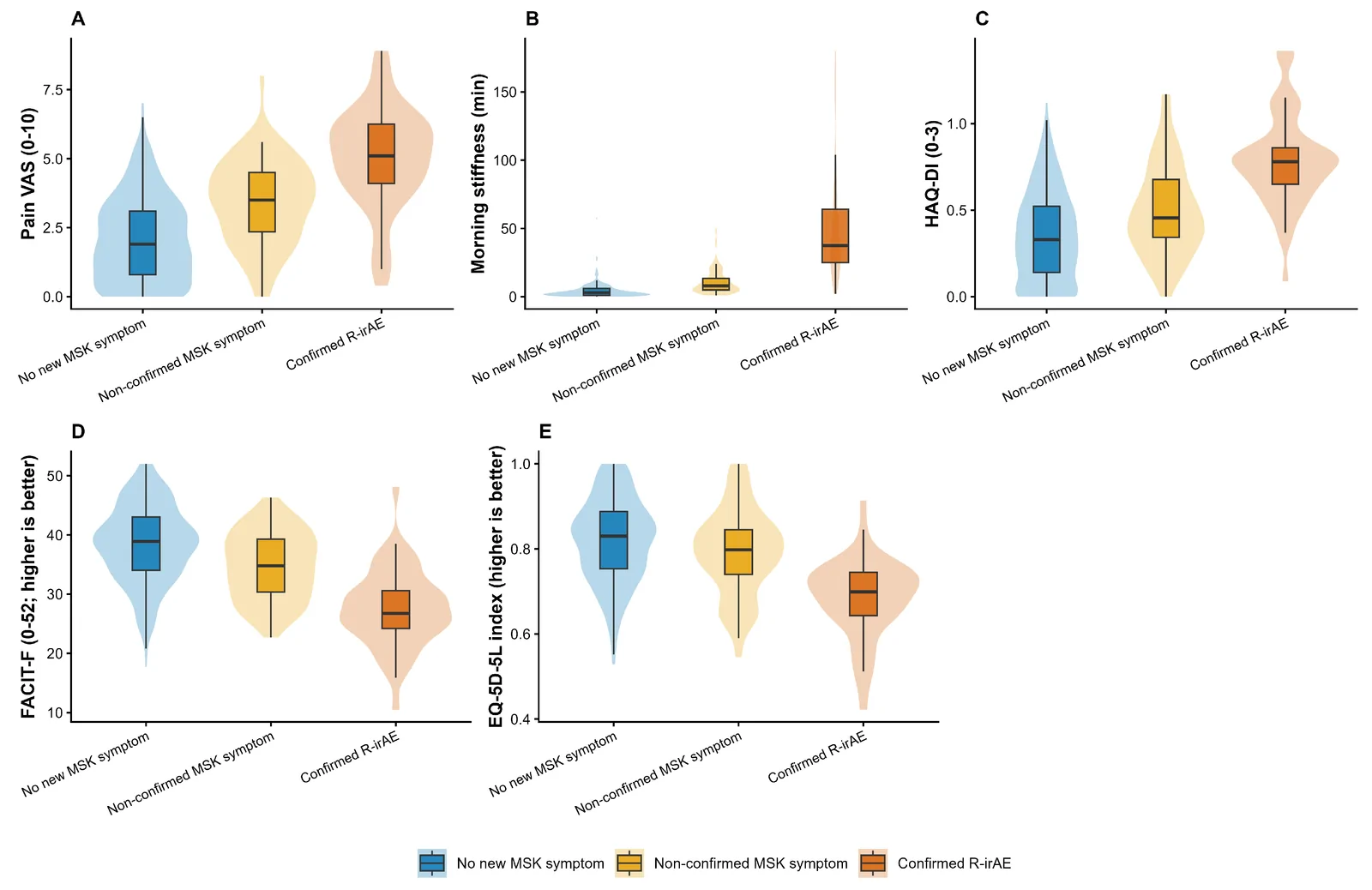
Individual patient-reported outcomes across post-ICI musculoskeletal symptom groups. (**A**) Pain visual analogue scale (VAS, 0–10); (**B**) morning stiffness duration (min); (**C**) Health Assessment Questionnaire Disability Index (HAQ-DI, 0–3); (**D**) Functional Assessment of Chronic Illness Therapy-Fatigue (FACIT-F, 0–52); (**E**) EQ-5D-5L index. Higher pain VAS, morning stiffness, and HAQ-DI indicate worse status, whereas higher FACIT-F and EQ-5D-5L indicate better status. ICI, immune checkpoint inhibitor; MSK, musculoskeletal; R-irAE, rheumatic immune-related adverse event.

**Table 1:** Baseline characteristics of lung cancer patients treated with immune checkpoint inhibitors.

Characteristic	Overall *N* = 768	No Confirmed R-irAE *N* = 702	Confirmed R-irAE *N* = 66	*p*-Value
Age, years	63.0 (57.0, 69.0)	63.0 (57.0, 69.0)	65.0 (56.0, 70.0)	0.893
Sex				0.001
Female	299 (38.9%)	261 (37.2%)	38 (57.6%)	
Male	469 (61.1%)	441 (62.8%)	28 (42.4%)	
Body mass index, kg/m^2^	23.1 (21.3, 25.5)	23.1 (21.3, 25.4)	23.7 (21.1, 26.1)	0.684
Smoking status				0.246
Never	345 (44.9%)	311 (44.3%)	34 (51.5%)	
Former	258 (33.6%)	235 (33.5%)	23 (34.8%)	
Current	165 (21.5%)	156 (22.2%)	9 (13.6%)	
ECOG performance status				0.447
0	222 (28.9%)	199 (28.3%)	23 (34.8%)	
1	413 (53.8%)	377 (53.7%)	36 (54.5%)	
2	121 (15.8%)	114 (16.2%)	7 (10.6%)	
3	12 (1.6%)	12 (1.7%)	0 (0.0%)	
Histology				0.515
Adenocarcinoma	515 (67.1%)	467 (66.5%)	48 (72.7%)	
Other NSCLC	33 (4.3%)	29 (4.1%)	4 (6.1%)	
Small-cell	67 (8.7%)	63 (9.0%)	4 (6.1%)	
Squamous	153 (19.9%)	143 (20.4%)	10 (15.2%)	
Stage group				0.525
I–II	38 (4.9%)	33 (4.7%)	5 (7.6%)	
III/limited	172 (22.4%)	157 (22.4%)	15 (22.7%)	
IV/extensive	558 (72.7%)	512 (72.9%)	46 (69.7%)	
Bone metastasis	154 (20.1%)	141 (20.1%)	13 (19.7%)	0.940
Brain metastasis	105 (13.7%)	96 (13.7%)	9 (13.6%)	0.993
PD-L1 TPS				0.429
<1%	163 (21.2%)	149 (21.2%)	14 (21.2%)	
1–49%	198 (25.8%)	184 (26.2%)	14 (21.2%)	
≥50%	173 (22.5%)	153 (21.8%)	20 (30.3%)	
Unknown	234 (30.5%)	216 (30.8%)	18 (27.3%)	
Actionable driver status				0.783
Negative	609 (79.3%)	558 (79.5%)	51 (77.3%)	
Actionable driver	74 (9.6%)	68 (9.7%)	6 (9.1%)	
Unknown	85 (11.1%)	76 (10.8%)	9 (13.6%)	
Treatment setting/line				0.223
Consolidation after CRT	78 (10.2%)	71 (10.1%)	7 (10.6%)	
First-line advanced	399 (52.0%)	363 (51.7%)	36 (54.5%)	
Neoadjuvant/adjuvant	45 (5.9%)	38 (5.4%)	7 (10.6%)	
Second/later line	246 (32.0%)	230 (32.8%)	16 (24.2%)	
ICI regimen				0.481
Dual ICI	21 (2.7%)	19 (2.7%)	2 (3.0%)	
ICI + anti-angiogenic	76 (9.9%)	71 (10.1%)	5 (7.6%)	
ICI + chemotherapy	418 (54.4%)	375 (53.4%)	43 (65.2%)	
ICI + radiotherapy/CRT	89 (11.6%)	84 (12.0%)	5 (7.6%)	
ICI monotherapy	164 (21.4%)	153 (21.8%)	11 (16.7%)	
ICI class				0.666
PD-L1	160 (20.8%)	149 (21.2%)	11 (16.7%)	
PD-1	587 (76.4%)	534 (76.1%)	53 (80.3%)	
PD-1 + CTLA-4	21 (2.7%)	19 (2.7%)	2 (3.0%)	
Baseline autoimmune history				<0.001
Autoimmune thyroiditis	10 (1.3%)	6 (0.9%)	4 (6.1%)	
Inflammatory bowel disease	7 (0.9%)	3 (0.4%)	4 (6.1%)	
No autoimmune disease	732 (95.3%)	677 (96.4%)	55 (83.3%)	
Psoriasis	10 (1.3%)	9 (1.3%)	1 (1.5%)	
Rheumatoid arthritis/Sjögren/SpA	9 (1.2%)	7 (1.0%)	2 (3.0%)	
Baseline CRP, mg/L	9.3 (6.1, 15.0)	9.1 (5.8, 14.7)	12.3 (7.5, 17.9)	0.003
Unknown	103	91	12	
Baseline eosinophils, ×10^9^/L	0.1 (0.1, 0.2)	0.1 (0.1, 0.2)	0.1 (0.1, 0.2)	0.922
Unknown	38	35	3	

Values are median (IQR) or *n* (%). Post-baseline variables, including PFS and other irAEs, were intentionally excluded. R-irAE, rheumatic immune-related adverse event; ICI, immune checkpoint inhibitor; CRP, C-reactive protein; ECOG, Eastern Cooperative Oncology Group; NSCLC, non-small-cell lung cancer; TPS, tumor proportion score; CRT, chemoradiotherapy; SpA, spondyloarthritis. *p* values were calculated using Wilcoxon rank-sum tests, Pearson chi-square tests, or Fisher exact tests, as appropriate; PD-1, programmed cell death protein 1; PD-L1, programmed death-ligand 1; CTLA-4, cytotoxic T-lymphocyte-associated protein 4.

**Table 2:** Clinical spectrum, onset, and management of confirmed rheumatic immune-related adverse events.

R-irAE Phenotype (*n*)	Onset, Days, Median (IQR)	Grade ≥ 3	Formal Referral	Systemic Steroid	csDMARD	Temporary ICI Hold Only	Permanent ICI Discontinuation	Improved/Resolved
Inflammatory arthritis (*n* = 22)	96 (61, 133)	5 (22.7%)	19 (86.4%)	12 (54.5%)	3 (13.6%)	10 (45.5%)	0 (0.0%)	21 (95.5%)
Inflammatory arthralgia (*n* = 15)	91 (37, 138)	1 (6.7%)	10 (66.7%)	7 (46.7%)	2 (13.3%)	4 (26.7%)	2 (13.3%)	13 (86.7%)
PMR-like syndrome (*n* = 13)	108 (65, 121)	3 (23.1%)	11 (84.6%)	9 (69.2%)	3 (23.1%)	2 (15.4%)	2 (15.4%)	12 (92.3%)
Sicca syndrome (*n* = 8)	227 (87, 302)	0 (0.0%)	4 (50.0%)	2 (25.0%)	3 (37.5%)	3 (37.5%)	0 (0.0%)	8 (100.0%)
Myositis (*n* = 4)	28 (17, 38)	1 (25.0%)	3 (75.0%)	3 (75.0%)	2 (50.0%)	2 (50%)	1 (25.0%)	4 (100.0%)
Spondyloarthritis-like (*n* = 2)	69 (56, 82)	0 (0.0%)	2 (100.0%)	2 (100.0%)	0 (0.0%)	0 (0.0%)	0 (0.0%)	2 (100.0%)
Vasculitis/overlap (*n* = 2)	111 (88, 134)	0 (0.0%)	2 (100.0%)	1 (50.0%)	1 (50.0%)	0 (0.0%)	0 (0.0%)	2 (100.0%)

Denominators are patients within each phenotype. Formal rheumatology referral denotes referral during routine clinical care and is distinct from rheumatologist adjudication of R-irAE status. Temporary ICI hold and permanent discontinuation are reported separately and are not mutually exclusive. No patient received a biologic DMARD. In the sicca subgroup, all eight patients had documented symptomatic improvement, but none had documented complete resolution; the three patients who received csDMARDs did so for concomitant inflammatory arthralgia or arthritis rather than for sicca itself. DMARD, disease-modifying antirheumatic drug; ICI, immune checkpoint inhibitor; PMR, polymyalgia rheumatica.

**Table 3:** Individual patient-reported outcomes in the nested questionnaire cohort.

Panel A. Descriptive individual patient-reported measures.				
Measure	No new MSK symptom *N* = 318	Non-confirmed MSK symptom *N* = 68	Confirmed R-irAE *N* = 51	*p*-value
Pain VAS, 0–10	1.9 (0.8, 3.1) *n* = 315	3.5 (2.3, 4.5) *n* = 67	5.1 (4.0, 6.3) *n* = 51	<0.001
Morning stiffness, min	3.0 (1.0, 6.0) *n* = 311	8.0 (5.0, 14.0) *n* = 63	37.5 (25.0, 64.0) *n* = 50	<0.001
HAQ-DI, 0–3	0.33 (0.14, 0.52) *n* = 312	0.45 (0.34, 0.68) *n* = 66	0.78 (0.65, 0.86) *n* = 49	<0.001
FACIT-F, 0–52	38.9 (34.0, 43.0) *n* = 308	34.8 (30.3, 39.3) *n* = 66	26.8 (24.1, 30.6) *n* = 48	<0.001
EQ-5D-5L index	0.830 (0.753, 0.888) *n* = 313	0.798 (0.740, 0.845) *n* = 65	0.699 (0.643, 0.745) *n* = 49	<0.001
Panel B. Adjusted comparisons for individual patient-reported outcomes.				
Outcome (model *N*)	Contrast		Adjusted mean difference (95% CI)	Holm-adjusted *p*-value
Pain VAS, 0–10 (*N* = 433)	Confirmed R-irAE vs. no new MSK symptom		3.1 (2.5 to 3.6)	<0.001
Pain VAS, 0–10 (*N* = 433)	Confirmed R-irAE vs. non-confirmed MSK symptom		1.8 (1.2 to 2.4)	<0.001
Morning stiffness, min (*N* = 424)	Confirmed R-irAE vs. no new MSK symptom		46.5 (35.4 to 57.6)	<0.001
Morning stiffness, min (*N* = 424)	Confirmed R-irAE vs. non-confirmed MSK symptom		40.5 (29.1 to 51.9)	<0.001
HAQ-DI, 0–3 (*N* = 427)	Confirmed R-irAE vs. no new MSK symptom		0.45 (0.37 to 0.54)	<0.001
HAQ-DI, 0–3 (*N* = 427)	Confirmed R-irAE vs. non-confirmed MSK symptom		0.30 (0.20 to 0.41)	<0.001
FACIT-F, 0–52 (*N* = 422)	Confirmed R-irAE vs. no new MSK symptom		−11.5 (−13.5 to −9.4)	<0.001
FACIT-F, 0–52 (*N* = 422)	Confirmed R-irAE vs. non-confirmed MSK symptom		−7.8 (−10.2 to −5.4)	<0.001
EQ-5D-5L index (*N* = 427)	Confirmed R-irAE vs. no new MSK symptom		−0.144 (−0.175 to −0.114)	<0.001
EQ-5D-5L index (*N* = 427)	Confirmed R-irAE vs. non-confirmed MSK symptom		−0.117 (−0.155 to −0.080)	<0.001

## Data Availability

The data presented in this study are available on request from the corresponding author due to ethical and privacy restrictions on hospital clinical data. De-identified analysis outputs may be available subject to institutional approval.

## Supplementary Materials

The following supporting information can be downloaded at https://www.mdpi.com/article/10.3390/curroncol33100603/s1, Supplementary Table S1A. Baseline missingness profile; Supplementary Table S1B. Availability of R-irAE workup variables; Supplementary Table S2. Questionnaire response-bias assessment; Supplementary Table S3. Unadjusted tumor outcomes by confirmed R-irAE status; Supplementary Table S4. Multiple-imputation core risk model for confirmed R-irAE; Supplementary Table S5. Risk-model sensitivity analyses; Supplementary Table S6. Detailed R-irAE workup and management by phenotype; Supplementary Table S7. Composite symptom-burden weighting sensitivity analysis; Supplementary Table S8. Autoantibody testing by musculoskeletal symptom group; Supplementary Table S9. IPW sensitivity analyses for individual patient-reported outcomes; Supplementary Table S10. Exploratory PFS models; Supplementary Figure S1. Multiple-imputation core risk factors for confirmed R-irAE; Supplementary Figure S2. Exploratory landmark progression-free survival by early R-irAE status.
