# Supplementary material for: Patient-Reported Burden and Clinical Spectrum of Rheumatic Immune-Related Adverse Events After Immune Checkpoint Inhibitor Therapy in Lung Cancer: A Real-World Cohort Study
Source: Curr Oncol. 2026 Oct 7;33(10):603. doi: 10.3390/curroncol33100603 (PMC13703647; doi:10.3390/curroncol33100603)
Supplement: Supplementary file 1 [file curroncol-33-00603-s001.zip › curroncol-4444880-supplementary.pdf]

Supplementary Materials

Patient-Reported Burden and Clinical Spectrum of Rheumatic Immune-Related Adverse Events after Immune Checkpoint Inhibitor Therapy in Lung Cancer: A Real-World Cohort Study

Supplementary Table S1A. Baseline missingness profile

Variable	Missing/unknown n	Missing/unknown %
PD-L1 TPS group	234	30.5
Driver status	85	11.1
Baseline CRP, mg/L	103	13.4
Baseline ESR, mm/h	266	34.6
Baseline LDH, U/L	62	8.1
Baseline eosinophils, $\times 10^9$/L	38	4.9

Baseline missingness among all 768 patients. PD-L1 TPS and driver status use unknown categories because absence of testing or insufficient tissue is clinically informative in real-world data.

Supplementary Table S1B. Availability of R-irAE workup variables

Measure	Denominator	Denominator n	Available/tested n	Available/tested %
Formal rheumatology referral status	Patients with new MSK symptom after ICI	177	177	100.0
Symptom attribution	Patients with new MSK symptom after ICI	177	177	100.0
R-irAE onset date	Confirmed R-irAE cases	66	66	100.0
CTCAE grade	Confirmed R-irAE cases	66	66	100.0
Rheumatology assessment delay	Confirmed R-irAE cases	66	66	100.0
Peak CRP	Confirmed R-irAE cases	66	66	100.0
Peak ESR	Confirmed R-irAE cases	66	66	100.0
CK checked	Confirmed R-irAE cases	66	22	33.3
Troponin checked	Myositis cases	4	4	100.0
ANA tested	Confirmed R-irAE cases	66	58	87.9
RF tested	Confirmed R-irAE cases	66	56	84.8
Anti-CCP tested	Confirmed R-irAE cases	66	53	80.3
SSA/SSB tested	Confirmed R-irAE cases	66	21	31.8

Availability of R-irAE workup variables is shown within clinically relevant denominators, not the full parent cohort. Formal rheumatology referral and symptom attribution are available for all 177 patients with new MSK symptoms after ICI. Formal referral denotes routine-care referral and is distinct from rheumatologist adjudication. CK and troponin were assessed within clinically relevant contexts. MSK, musculoskeletal; CK, creatine kinase; CTCAE, Common Terminology Criteria for Adverse Events.

Supplementary Table S2. Questionnaire response-bias assessment

Characteristic	Eligible nonresponder<br>N = 325	Responder<br>N = 437	p-value
Age, years	62.0 (57.0, 69.0)	63.0 (57.0, 69.0)	0.464
Sex			0.109
Female	116 (35.7%)	181 (41.4%)	
Male	209 (64.3%)	256 (58.6%)	
ECOG PS			0.382
0	95 (29.2%)	126 (28.8%)	
1	170 (52.3%)	239 (54.7%)	
2	52 (16.0%)	68 (15.6%)	
3	8 (2.5%)	4 (0.9%)	
Stage group			0.872
I-II	17 (5.2%)	21 (4.8%)	
III/limited	74 (22.8%)	94 (21.5%)	
IV/extensive	234 (72.0%)	322 (73.7%)	
Confirmed R-irAE			<0.001
No confirmed R-irAE	310 (95.4%)	386 (88.3%)	
Confirmed R-irAE	15 (4.6%)	51 (11.7%)	
New MSK symptom after ICI	57 (17.5%)	119 (27.2%)	0.002
Observed PFS time, months	8.7 (4.2, 15.4)	9.0 (4.7, 16.1)	0.577
Death by administrative follow-up	245 (75.4%)	302 (69.1%)	0.057

Values are median (IQR) or n (%). The comparison is restricted to 762 patients who were eligible for questionnaire assessment; 437 responded and 325 did not. P values were calculated using Wilcoxon rank-sum tests or Pearson chi-square tests. Stabilized and truncated IPW sensitivity analyses for the individual patient-reported outcomes are shown in Supplementary Table S9. Questionnaire eligibility required being alive at the questionnaire assessment window.

Supplementary Table S3. Unadjusted tumor outcomes by confirmed R-irAE status

R-irAE status	n	Objective response	Disease control	PFS, months, median (IQR)	PFS event	OS, months, median (IQR)	Death
No confirmed R-irAE	702	314 (44.7%)	589 (83.9%)	8.4 (4.1, 15.3)	647 (92.2%)	20.9 (14.4, 28.5)	511 (72.8%)
Confirmed R-irAE	66	35 (53.0%)	59 (89.4%)	12.2 (8.1, 19.1)	48 (72.7%)	19.9 (13.3, 26.3)	42 (63.6%)

Unadjusted cancer outcomes; interpret survival cautiously because R-irAE is post-baseline. These results are exploratory and should not be used to claim that R-irAE independently predicts tumor response.

Supplementary Table S4. Multiple-imputation core risk model for confirmed R-irAE

Variable	Effect	Estimate (95% CI)	p-value
Female sex	Adjusted OR	2.21 (1.31 to 3.74)	0.003
Age, per year	Adjusted OR	1.00 (0.97 to 1.03)	0.955
ECOG PS >=2	Adjusted OR	0.53 (0.23 to 1.22)	0.133
ICI + chemotherapy	Adjusted OR	1.57 (0.91 to 2.72)	0.104
Baseline CRP >10 mg/L	Adjusted OR	2.08 (1.14 to 3.81)	0.017
Pre-existing autoimmune disease	Adjusted OR	5.24 (2.37 to 11.57)	<0.001

Primary multivariable logistic regression model for rheumatologist-confirmed R-irAE. Missing baseline CRP (103/768, 13.4%) was handled using multiple imputation by chained equations with 50 imputations; continuous CRP was imputed using predictive mean matching and the >10 mg/L indicator was derived within each imputed dataset. Estimates were pooled using Rubin's rules. The model includes six baseline covariates to limit overfitting with 66 confirmed events. The CRP imputation model included the R-irAE outcome, all non-CRP core-model covariates, and auxiliary baseline variables (body mass index, stage IV/extensive disease, bone and brain metastases, albumin, hemoglobin, neutrophil, lymphocyte, and platelet counts).

Supplementary Table S5. Risk-model sensitivity analyses

Analysis	Variable	Estimate (95% CI)	P value
Multiple-imputation logistic model	Female sex	2.21 (1.31 to 3.74)	0.003
Multiple-imputation logistic model	Age, per year	1.00 (0.97 to 1.03)	0.955
Multiple-imputation logistic model	ECOG PS >=2	0.53 (0.23 to 1.22)	0.133
Multiple-imputation logistic model	ICI + chemotherapy	1.57 (0.91 to 2.72)	0.104
Multiple-imputation logistic model	Baseline CRP >10 mg/L	2.08 (1.14 to 3.81)	0.017
Multiple-imputation logistic model	Pre-existing autoimmune disease	5.24 (2.37 to 11.57)	<0.001
Complete-case logistic model	Female sex	2.00 (1.12 to 3.58)	0.019
Complete-case logistic model	Age, per year	1.00 (0.97 to 1.03)	0.978
Complete-case logistic model	ECOG PS >=2	0.56 (0.21 to 1.29)	0.210
Complete-case logistic model	ICI + chemotherapy	1.69 (0.93 to 3.15)	0.090
Complete-case logistic model	Baseline CRP >10 mg/L	2.21 (1.23 to 4.08)	0.009
Complete-case logistic model	Pre-existing autoimmune disease	4.87 (1.97 to 11.28)	<0.001
Complete-case Firth logistic model	Female sex	1.97 (1.12 to 3.51)	0.019
Complete-case Firth logistic model	Age, per year	1.00 (0.97 to 1.03)	0.989
Complete-case Firth logistic model	ECOG PS >=2	0.60 (0.23 to 1.34)	0.227
Complete-case Firth logistic model	ICI + chemotherapy	1.65 (0.92 to 3.06)	0.091
Complete-case Firth logistic model	Baseline CRP >10 mg/L	2.16 (1.22 to 3.95)	0.008
Complete-case Firth logistic model	Pre-existing autoimmune disease	4.84 (2.01 to 10.95)	<0.001
Definite-only outcome, Firth logistic model	Female sex	1.67 (0.87 to 3.21)	0.121
Definite-only outcome, Firth logistic model	Age, per year	1.00 (0.97 to 1.04)	0.944
Definite-only outcome, Firth logistic model	ECOG PS >=2	0.56 (0.18 to 1.41)	0.235
Definite-only outcome, Firth logistic model	ICI + chemotherapy	1.67 (0.86 to 3.39)	0.133
Definite-only outcome, Firth logistic model	Baseline CRP >10 mg/L	2.39 (1.23 to 4.85)	0.010
Definite-only outcome, Firth logistic model	Pre-existing autoimmune disease	4.87 (1.85 to 11.67)	0.002

Sensitivity analyses include the multiple-imputation primary model, complete-case conventional logistic regression, complete-case Firth logistic regression, and a definite-only R-irAE Firth model. The female-sex association was attenuated in the definite-only analysis, whereas elevated baseline CRP and pre-existing autoimmune disease remained associated with the outcome.

Supplementary Table S6. Detailed R-irAE workup and management by phenotype

R-irAE phenotype	n	Assessment delay, days, median (IQR)	Peak CRP, mg/L, median (IQR)	Peak ESR, mm/h, median (IQR)	Temporary ICI hold only	Permanent ICI discontinuation	NSAID/analgesic	Intra-articular steroid
Inflammatory arthritis	22	21 (13, 32)	25.6 (18.3, 40.3)	48.0 (44.0, 63.0)	10/22 (45.5%)	0/22 (0.0%)	16/22 (72.7%)	2/22 (9.1%)
Inflammatory arthralgia	15	18 (11, 36)	20.0 (15.4, 36.1)	35.0 (25.0, 57.0)	4/15 (26.7%)	2/15 (13.3%)	10/15 (66.7%)	0/15 (0.0%)
PMR-like syndrome	13	18 (15, 26)	35.6 (17.4, 48.4)	51.0 (45.0, 57.0)	2/13 (15.4%)	2/13 (15.4%)	10/13 (76.9%)	0/13 (0.0%)
Sicca syndrome	8	23 (15, 78)	12.1 (9.2, 14.4)	41.0 (31.5, 50.0)	3/8 (37.5%)	0/8 (0.0%)	6/8 (75.0%)	0/8 (0.0%)
Myositis	4	36 (24, 41)	32.0 (24.3, 36.3)	59.0 (51.5, 68.0)	2/4 (50.0%)	1/4 (25.0%)	3/4 (75.0%)	0/4 (0.0%)
Spondyloarthritis-like	2	14 (13, 14)	23.2 (19.7, 26.7)	26.5 (5.0, 48.0)	0/2 (0.0%)	0/2 (0.0%)	2/2 (100.0%)	0/2 (0.0%)
Vasculitis/overlap	2	23 (22, 24)	26.9 (22.0, 31.7)	64.0 (45.0, 83.0)	0/2 (0.0%)	0/2 (0.0%)	2/2 (100.0%)	0/2 (0.0%)

Detailed workup and management among confirmed R-irAE cases. Assessment delay refers to the interval between first symptom documentation and rheumatology assessment/adjudication and should not be interpreted as formal-referral delay. Temporary ICI hold and permanent ICI discontinuation are mutually exclusive. Patients who initially had ICI treatment held and subsequently permanently discontinued ICI because of R-irAE were classified in the permanent-discontinuation category only. In the eight sicca cases, all had documented symptomatic improvement during follow-up and none had documented complete resolution; hydroxychloroquine was used in two patients and methotrexate in one for concomitant inflammatory arthralgia/arthritis. In the four myositis cases, no myocarditis or myasthenic syndrome was documented. ESR, erythrocyte sedimentation rate; CRP, C-reactive protein; ICI, immune checkpoint inhibitor; NSAID, non-steroidal anti-inflammatory drug.

Supplementary Table S7. Composite symptom-burden weighting sensitivity analysis

Scenario	Weights Pain/Disability/Fatigue/Stiffness	Contrast	N	Adjusted mean difference (95% CI)	p-value
Original analysis-defined weights	0.35/0.25/0.20/0.20	Confirmed R-irAE vs no new MSK symptom	396	26.9 (23.4 to 30.3)	<0.001
Original analysis-defined weights	0.35/0.25/0.20/0.20	Confirmed R-irAE vs non-confirmed MSK symptom	396	18.4 (14.5 to 22.4)	<0.001
Equal weights	0.25/0.25/0.25/0.25	Confirmed R-irAE vs no new MSK symptom	396	26.8 (23.4 to 30.2)	<0.001
Equal weights	0.25/0.25/0.25/0.25	Confirmed R-irAE vs non-confirmed MSK symptom	396	19.0 (15.2 to 22.8)	<0.001
Pain-emphasis	0.40/0.20/0.20/0.20	Confirmed R-irAE vs no new MSK symptom	396	27.6 (24.1 to 31.2)	<0.001
Pain-emphasis	0.40/0.20/0.20/0.20	Confirmed R-irAE vs non-confirmed MSK symptom	396	18.8 (14.7 to 22.9)	<0.001
Disability-emphasis	0.20/0.40/0.20/0.20	Confirmed R-irAE vs no new MSK symptom	396	24.5 (21.5 to 27.5)	<0.001
Disability-emphasis	0.20/0.40/0.20/0.20	Confirmed R-irAE vs non-confirmed MSK symptom	396	17.3 (13.8 to 20.8)	<0.001
Fatigue-emphasis	0.20/0.20/0.40/0.20	Confirmed R-irAE vs no new MSK symptom	396	26.0 (22.7 to 29.3)	<0.001
Fatigue-emphasis	0.20/0.20/0.40/0.20	Confirmed R-irAE vs non-confirmed MSK symptom	396	18.2 (14.5 to 22.0)	<0.001
Stiffness-emphasis	0.20/0.20/0.20/0.40	Confirmed R-irAE vs no new MSK symptom	396	28.9 (25.0 to 32.9)	<0.001
Stiffness-emphasis	0.20/0.20/0.20/0.40	Confirmed R-irAE vs non-confirmed MSK symptom	396	21.7 (17.4 to 26.0)	<0.001

Secondary exploratory analysis among 396 questionnaire responders with complete data for pain VAS, HAQ-DI, FACIT-F, and morning stiffness. The original analysis-defined weights were compared with equal weighting and one-domain-emphasis schemes. The composite is not a validated standalone instrument; the sensitivity analysis evaluates whether the observed group contrast depends on the original weighting recipe.

Supplementary Table S8. Autoantibody testing by musculoskeletal symptom group

Test	Non-confirmed MSK symptom: Tested	Confirmed R-irAE: Tested	Non-confirmed MSK symptom: Positive among tested	Confirmed R-irAE: Positive among tested
ANA	24/111 (21.6%)	58/66 (87.9%)	6/24 (25.0%)	21/58 (36.2%)
Rheumatoid factor	14/111 (12.6%)	56/66 (84.8%)	1/14 (7.1%)	7/56 (12.5%)
Anti-CCP	14/111 (12.6%)	53/66 (80.3%)	1/14 (7.1%)	2/53 (3.8%)
SSA/SSB	0/111 (0.0%)	21/66 (31.8%)	NE	2/21 (9.5%)

Autoantibody testing was clinically driven rather than protocolized. Tested proportions therefore differ substantially between the non-confirmed MSK symptom and confirmed R-irAE groups. Positivity is reported only among tested patients and should not be interpreted as a prevalence estimate. NE, not estimable because no SSA/SSB testing was recorded in the non-confirmed MSK symptom group. No formal between-group hypothesis test of antibody positivity was performed because testing was nonprotocolized and test availability differed markedly between groups.

Supplementary Table S9. IPW sensitivity analyses for individual patient-reported outcomes

Outcome	Contrast	N	IPW-adjusted mean difference (95% CI)	P value	Holm-adjusted P value
Pain VAS, 0-10	Confirmed R-irAE vs no new MSK symptom	433	3.1 (2.5 to 3.6)	<0.001	<0.001
Pain VAS, 0-10	Confirmed R-irAE vs non-confirmed MSK symptom	433	1.8 (1.2 to 2.4)	<0.001	<0.001
Morning stiffness, min	Confirmed R-irAE vs no new MSK symptom	424	46.4 (35.7 to 57.2)	<0.001	<0.001
Morning stiffness, min	Confirmed R-irAE vs non-confirmed MSK symptom	424	40.2 (29.1 to 51.3)	<0.001	<0.001
HAQ-DI, 0-3	Confirmed R-irAE vs no new MSK symptom	427	0.46 (0.37 to 0.54)	<0.001	<0.001
HAQ-DI, 0-3	Confirmed R-irAE vs non-confirmed MSK symptom	427	0.30 (0.20 to 0.40)	<0.001	<0.001
FACIT-F, 0-52	Confirmed R-irAE vs no new MSK symptom	422	-11.5 (-13.5 to -9.5)	<0.001	<0.001
FACIT-F, 0-52	Confirmed R-irAE vs non-confirmed MSK symptom	422	-7.8 (-10.1 to -5.5)	<0.001	<0.001
EQ-5D-5L index	Confirmed R-irAE vs no new MSK symptom	427	-0.145 (-0.174 to -0.115)	<0.001	<0.001
EQ-5D-5L index	Confirmed R-irAE vs non-confirmed MSK symptom	427	-0.118 (-0.154 to -0.082)	<0.001	<0.001

IPW analyses were restricted to questionnaire-eligible patients. The response model included age, sex, ECOG PS ≥2, stage IV/extensive disease, bone metastasis, pre-existing autoimmune disease, confirmed R-irAE, and new MSK symptoms after ICI. Stabilized weights were truncated at the 1st and 99th percentiles. Holm-adjusted p values account for the 10 reported contrasts.

Supplementary Table S10. Exploratory PFS models

Analysis	Effect	Estimate (95% CI)	P value
3-month landmark PFS model	Adjusted HR	0.79 (0.51 to 1.22)	0.283
Time-dependent PFS model	Adjusted HR	0.75 (0.56 to 1.00)	0.049

The 3-month landmark and time-dependent Cox models are exploratory. Adjusted models include age, sex, ECOG PS, stage IV/extensive disease, PD-L1 TPS ≥50%, actionable driver status, and ICI plus chemotherapy. These analyses should not be interpreted as evidence that R-irAE independently predicts tumor benefit.

Supplementary Figure S1. Multiple-imputation core risk factors for confirmed R-irAE

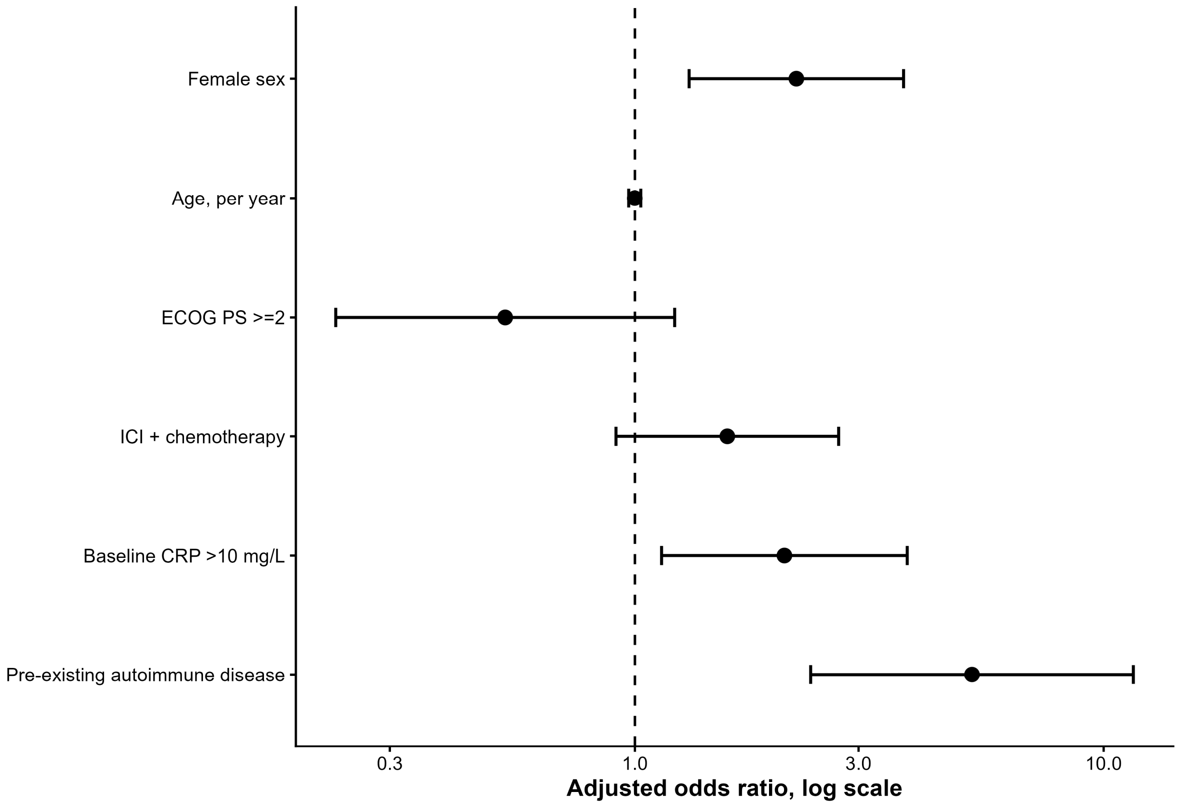

Legend. Points are pooled adjusted odds ratios and horizontal bars are 95% confidence intervals from the multiple-imputation core logistic regression model.

Supplementary Figure S2. Exploratory landmark progression-free survival by early R-irAE status

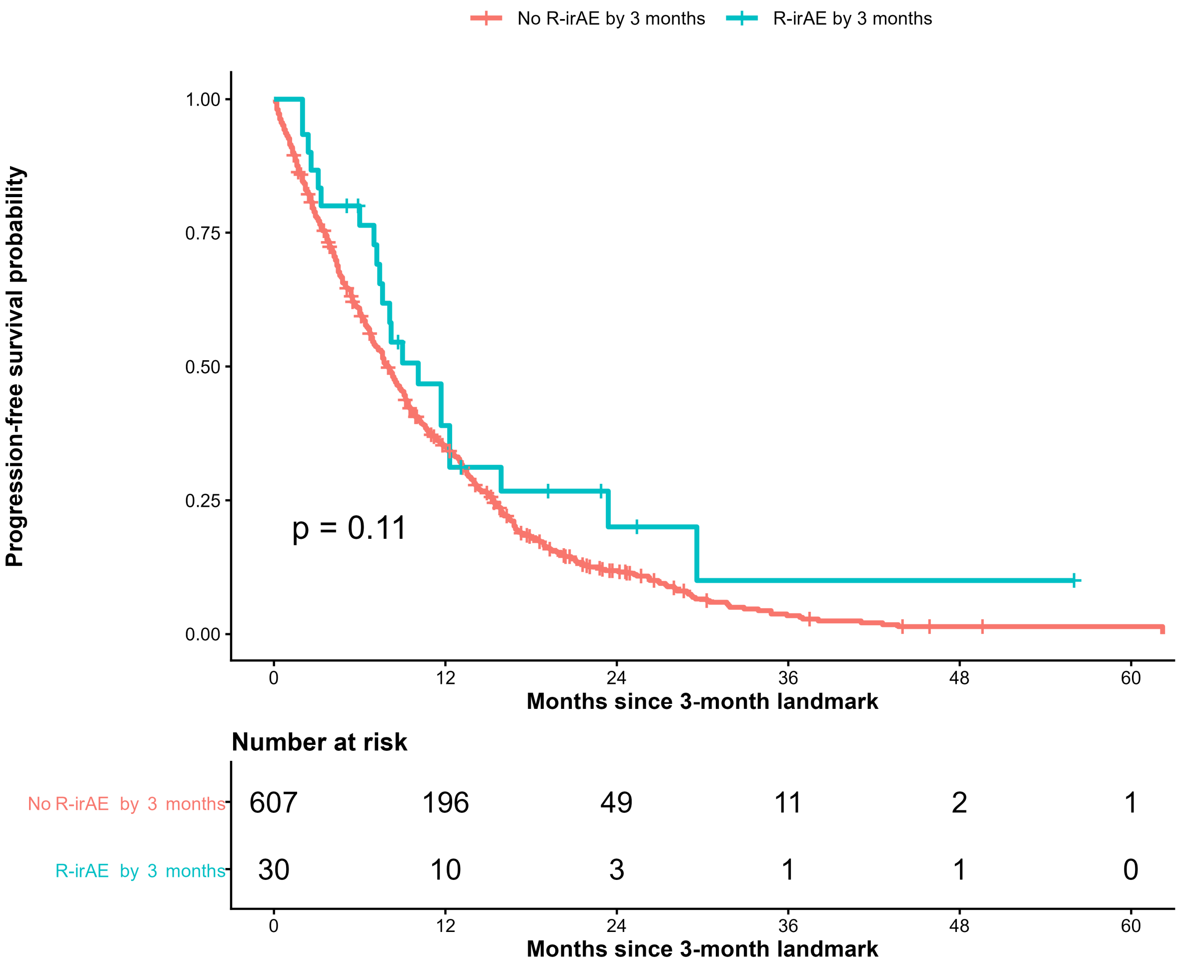

Legend. Landmark time is 3 months after ICI initiation. Patients with progression or follow-up shorter than the landmark are excluded to reduce immortal-time bias. The adjusted 3-month landmark Cox model is exploratory and should not be interpreted as evidence that R-irAE independently predicts tumor benefit.
